# Dynamic disconnection of the supplementary motor area after processing of dismissive biographic narratives

**DOI:** 10.1002/brb3.377

**Published:** 2015-09-14

**Authors:** Viola Borchardt, Anna L. Krause, Meng Li, Marie‐José van Tol, Liliana Ramona Demenescu, Anna Buchheim, Coraline D. Metzger, Catherine M. Sweeney‐Reed, Tobias Nolte, Anton R. Lord, Martin Walter

**Affiliations:** ^1^ Leibniz Institute for NeurobiologyMagdeburgGermany; ^2^ Clinical Affective Neuroimaging LaboratoryMagdeburgGermany; ^3^ Department of Psychiatry and PsychotherapyOtto von Guericke UniversityMagdeburgGermany; ^4^ Department of NeurologyOtto von Guericke UniversityMagdeburgGermany; ^5^ University of GroningenUniversity Medical Center GroningenNeuroimaging CenterGroningenthe Netherlands; ^6^ Institute of PsychologyUniversity of InnsbruckInnsbruckAustria; ^7^ Institute for Cognitive Neurology and Dementia Research (IKND)MagdeburgGermany; ^8^ Neurocybernetics and RehabilitationDepartment for Neurology and Stereotactic NeurosurgeryOtto von Guericke UniversityMagdeburgGermany; ^9^ Anna Freud CentreLondonUK; ^10^ Wellcome Trust Centre for NeuroimagingUniversity College of LondonLondonUK; ^11^ Center for Behavioral Brain Sciences (CBBS)MagdeburgGermany; ^12^ German Center for Neurodegenerative Diseases (DZNE)MagdeburgGermany

**Keywords:** Acoustic stimulation, adult attachment representation, cognitive affective neuroscience, fMRI, functional connectivity, graph theory, human social interactions, resting‐state

## Abstract

**Introduction:**

To understand the interplay between affective social information processing and its influence on mental states we investigated changes in functional connectivity (FC) patterns after audio exposure to emotional biographic narratives.

**Methods:**

While lying in the 7T MR scanner, 23 male participants listened to narratives of early childhood experiences of three persons, each having either a secure, dismissing, or preoccupied attachment representation. Directly after having listened to each of the prototypical narratives, participants underwent a 10‐minute resting‐state fMRI scan.

To study changes in FC patterns between experimental conditions, three post‐task conditions were compared to a baseline condition. Specific local alterations, as well as differences in connectivity patterns between distributed brain regions, were quantified using Network‐based statistics (NBS) and graph metrics.

**Results:**

Using NBS, a nine‐region subnetwork showing reduced FC after having listened to the dismissing narrative was identified. Of this subnetwork, only the left Supplementary Motor Area (SMA) exhibited a decrease in the nodal graph metrics degree and strength exclusively after listening to the dismissing narrative. No other region showed post‐task changes in nodal metrics. A post hoc analysis of dynamic characteristics of FC of the left SMA showed a significant decrease in the dismissing condition when compared with the other conditions in the first three minutes of the scan, but faded away in the two subsequent intervals the differences.

**Conclusions:**

Nodal metrics and NBS converge on reduced connectivity measures exclusively in left SMA in the dismissing condition, which may specifically reflect ongoing network changes underlying prolonged emotional reactivity to attachment‐related processing.

## Introduction

Areas of the brain are not only anatomically connected, but importantly also functionally coupled (Behrens and Sporns 2012). Functional brain networks, also called intrinsic functional connectivity networks (ICNs), do not consist of a fixed set of regions, but rather can be seen as temporally clustered communities exhibiting synchronized activation (Cohen et al. 2008; Sadaghiani et al. 2010).

Differing ICN patterns have been found in a healthy population during sleep, unconscious, and wakeful states (Horovitz et al. 2009; Vanhaudenhuyse et al. 2010). Furthermore, deviant ICN patterns have been associated with mood disorders (Wang et al. 2012; Whalley et al. 2012). ICNs have the ability to reconfigure their patterns of interconnections (typically clustered by coactivation or deactivation) according to specific demands of incoming cognitive tasks and stimuli (Smith et al. 2009). Understandably, this adaptive change in topology due to activation state varies over time.

Intrinsic changes in connectivity patterns occur across different levels of ongoing activity, are context‐dependent, and related to recent cognitive experiences (Waites et al. 2005; Tambini et al. 2012). Such flexibility in intrinsic functional architecture is necessary to appropriately evaluate fast‐changing external stimuli, and ultimately dynamically adjust personal appraisals shaping subsequent reactions (Hutchison et al. 2013).

Mood, defined as an emotional state prolonged in temporal duration, has the propensity to influence the subjective cognitive state and consequently ICNs, as shown by work of Harrison et al., who found that transient changes in functional connectivity (FC) emanate from experimentally controlled changes in subjective mood states of subjects without pathological mood disturbances (Harrison et al. 2008).

Changes in ICNs have not only been established immediately after a cognitive stimulus but, interestingly, also on a prolonged time scale:

Barnes et al. studied the brain's response to a cognitive performance (n‐back) task in a rest‐task‐rest design and identified carry‐over effects: The more demanding the task was, the slower the subsequent recovery of endogenous dynamics to pretask values. This shows that endogenous dynamics are relevant to the brain's response to exogenous stimulation and indicates that changes in the mental state lead to changes in brain connectivity (Barnes et al. 2009). Waites et al. demonstrated that FC maps, derived from a resting‐state scan acquired directly after a language task, capture a combination of static and transient synchrony between brain states (Waites et al. 2005). Moreover, mood and cognitive state of a person are strongly modulated by external social as well as emotional stimuli (Harrison et al. 2008). The perception of a situation, and thus also the brain's dynamic response to it, however, depend not only on current mood and cognitive load but also on personal experiences from the past, which have shaped how individuals react to social‐emotional stimuli (Davidson 1998). While throughout life, social bonds between people are established and maintained, especially experiences in early childhood are crucially responsible for social emotional functioning (Beck 1987, 2008). Attachment theory, a developmental psychological framework, suggests that interactions with attachment figures (e.g., parents), and the responses of the latter to the proximity‐seeking attempts of the child, will induce the formation of differential cognitive schemata for representation of the self and others, and for behavior and affect regulation in interpersonal relationships later on in life (Cassidy 2008; Nolte et al. 2011; Vrticka and Vuilleumier 2012). Schema activation, as explained, for instance, in the cognitive model of depression (Disner et al. 2011) can be seen as a psychopathologically relevant “carry‐over” effect of an environmental trigger.

Since personal representations of attachment are thought to have profound influences on individual responses to social affective cues, we strongly supposed that, firstly, interpersonal experience with attachment‐related content is a contributing factor to inducing significant changes in mental states, and that secondly, subsequent processing of social emotional information is influenced by individual predispositions. The aim of the study reported here was to investigate changes in brain network connectivity patterns after participants listened to three emotional biographical narratives of strangers (post‐task rs‐fMRI). The narratives/conditions are termed according to the attachment representation of the interviewee, namely secure, insecure‐preoccupied, and insecure‐dismissing.

We hypothesized that experimental activation leads to specific and prolonged changes in resting‐state ICN pattern. Furthermore, we speculated that particularly during processing of attachment‐related information with negative valence, such as included in the dismissing and preoccupied narratives used in our paradigm, processing and regulatory mechanisms may be altered when compared to the secure narrative, which may be seen as a rather neutral stimulus, since biographical material, irrespective of emotional quality, is presented in a coherent and contained manner.

Due to the relatively sparse literature pertaining to emotional reactivity and attachment, we chose a data‐driven framework to investigate changes in ICNs evoked by the task. We used the sophisticated method of graph theory to investigate potential differences in connectivity patterns between distributed brain regions during different experimental conditions. We opted for an analytic approach in which the brain is parcellated into many regions of interest (ROIs) extending over the whole brain. In contrast to seed‐based analytic approaches, this entails the advantage of not needing an a‐priori hypotheses. Benefitting from the post‐task resting‐state design and the data‐driven analysis approach, brain networks modulating processing of attachment‐related information might be uncovered that may not yet have been revealed by prior task‐based paradigms. Furthermore, with the help of nodal metrics, graph theory allows for the description of specific local properties of brain areas as well as their functional interplay in the network and can accordingly quantify local alterations between experimental conditions beyond their functional connectivity.

In a post hoc analysis, we additionally assessed dynamic changes of connectivity patterns to investigate the temporal characteristics of the ICN patterns.

## Methods

### Subjects

Twenty‐three healthy, male, right‐handed, native German speakers (mean age: 29.8, SD: 3.5), who were screened for psychiatric, neurological, or medical illnesses, were inclu‐ded. In addition to any psychiatric disorder, exclusion criteria contained the common exclusion criteria for Magnetic Resonance Imaging (MRI). The institutional review board of the University of Magdeburg, Germany, approved the study and all subjects provided written informed consent.

### Data acquisition

Resting‐state BOLD data were acquired in a 7T whole body MR system (Siemens, Erlangen, Germany) with a 32‐channel receiver coil, using an EPI sequence (TR 2.61 s, TE 22 ms, 240 time points, 50 slices, voxel size 1.6 mm isotropic). T1‐weighted anatomical reference data were imaged using 3D‐MPRAGE (1 mm isotropic resolution, TE 2.01 s, TI 1050 ms, TR 1700 ms, flip angle 5°). The subjects were instructed to simply lie still inside the scanner with their eyes closed, to think of nothing in particular and to stay awake. Motion was minimized using soft pads fitted over the ears and participants were given earplugs to minimize noise.

### Experimental design

**Figure 1:**
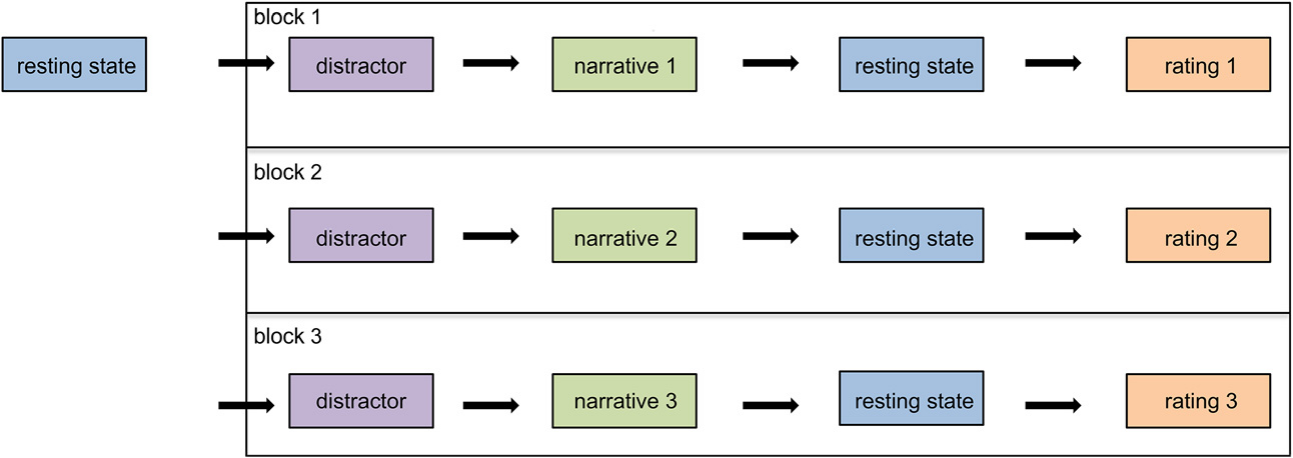
Course of tasks in the experimental design.

We conducted a repeated rest‐task‐rest design (Fig. 1) with a baseline resting‐state scan at the beginning followed by the three blocks. Each block consisted of four parts. First, the subjects were asked to perform easy calculations for 90 sec as a distractor task. Then, one of three narratives was presented, directly followed by a 10 min post‐task resting‐state scan, for which subjects were instructed to observe their feelings in response to the narrative. Afterward, subjects were asked to rate both their own feelings and the narrative with the questionnaire described below. This procedure was iterated thrice, with each narrative was presented once. To control for effects of presentation order, the order was randomized between subjects.

During the experiment, subjects listened to three nar‐ratives, characteristic of the secure, insecure‐dismissing, and insecure‐preoccupied attachment representations (Kirchmann et al. 2011). These narratives are prototypical excerpts of Question 3 and 4 of the Adult Attachment Interview (AAI) in which the interviewees were asked to describe their relationship with their father and mother during childhood with characteristic words and accompanying episodes to illustrate them. The interviewees were three patients with anxiety disorder classified into the following AAI categories: dismissing, preoccupied, and secure‐autonomous. To ensure anonymity and to avoid the impact of different voices, all transcripts were authentically read and recorded by Prof. Anna Buchheim. In previous studies of (Martin et al. 2007; and (Kirchmann et al. 2011), the length of the tapes differed from 3:27 min (insecure‐dismissing) to 8:51 min (insecure‐preoccupied). To avoid effects of tape duration, the tapes were shortened to a length of 4:58 min (insecure‐preoccupied) and 4:08 min (secure‐autonomous). Because it is important to preserve attachment‐specific speech characteristics, we ensured these were maintained during shortening of the audio stimuli (for further details see (Krause et al. 2015).

Due to an overlap in cognitive demands involved in processing of auditory stimuli, we expected extensive similarities between the post‐task resting‐state scans following different stimuli. Due to this, we expected general effects of narratives, regardless of emotional content, to be dependent between conditions. Therefore, results reported throughout this study speak more toward variations elicited by the affective content of the narratives than the effects of auditory processing in general.

To evaluate the effect of the narratives on mood and wellbeing of the participants, after each resting phase, participants answered 40 questions presented on the screen via button presses. However, these answers were not assessed in this study.

After the experiment, participants filled in German versions of the Temperament and Character Inventory (TCI) questionnaire (Cloninger et al. 1993), Childhood Trauma Questionnaire (CTQ) (Scher et al. 2001; Bernstein et al. 2003), and Beck's Depression Inventory (BDI) (Hautzinger et al. 2009). Before and after the experiments, subjects filled in the German version of the multidimensional mood questionnaire (MDBF) (Steyer et al. 1997; Hinz et al. 2012), which assesses mood, vigilance, and agitation.

### Data processing

Resting‐state functional connectivity data underwent standard preprocessing steps as implemented in SPM12 (RRID: nif‐0000‐00343) (Ashburner et al. 2012), namely slice time acquisition correction, realignment, coregistration, segmentation, and normalization. Using the DPARSF toolbox V2.3 (RRID: nlx_155735) (Yan and Zang 2010), the first 10 timepoints were removed to account for signal equilibrium and adaptation effects, temporal filtering was performed in the range 0.01–0.08 Hz, and nuisance covariate regression included a first‐order polynomial trend (detrending), using a six parameters rigid body head motion model, white matter, and CSF signals. Global mean regression and smoothing were not performed. We investigated head motion of the subjects with exclusion criteria of 1.0 mm and 1.0 degree in maximal head motion at a voxel size of 1.6 mm. One subject was excluded due to excessive movement.

Using DPARSFA, time courses of 104 predefined ROIs (Table S2) were extracted and connectivity matrices filled with pair‐wise Pearson correlation coefficients were created. These ROIs were based on the general brain parcellation of the AAL atlas (Tzourio‐Mazoyer et al. 2002). Additional parcellation was performed for the cingulate and insular subregions due to their functional segregation and specialization (Fig. S1) (Yu et al. 2011; Dou et al. 2013).

### Data analysis

#### Network‐based statistics

Brain network analysis was performed using methods of graph theory, where functional networks are represented as a graph of nodes (brain regions) connected by edges (functional connections). The functionality of an individual node is to a certain extent determined by the pattern of its interconnections (Bullmore and Sporns 2009).

Using graph theory, brain networks can be characterized on a topological, modular, and nodal level using specific metrics. A related approach called Network‐based statistics (NBS) can be applied even before graph networks are constructed. For NBS, information about functional connectivity between regions stored in the connectivity matrix is used. NBS is a method to identify a statistically significant cluster of connections in a brain network, that is, subnetworks, which may be associated with a changing psychological context. Specifically, subnetworks identified using a t‐statistic represents a subset of nodes sharing reduced functionally connected edges that, as a whole, are statistically unlikely to be due to random chance. To validate that the resulting network is not due to a random effect, group membership is resampled 5000 times, and the NBS attempts to identify significant subnetworks in these surrogate data. The returned network from the real data is statistically significant at a family wise error (FWE) corrected value of *P* < 0.05. However, the influence of individual components cannot be identified. Although the network needs to be considered as a whole, the extent of the returned subnetwork can be varied using a threshold parameter. This adjusts the extremity of deviation in a connection between groups required before it is considered for inclusion in the NBS result. By altering this primary t‐statistic threshold, any resulting significant cluster may be altered in size, although it should be noted that if altering the threshold creates a larger cluster, a smaller cluster found with a different threshold would be encompassed inside the larger one. This mentioned threshold is arbitrarily chosen and must not be confused with a threshold used to render the networks sparse, which will be used in later analyses. Because for NBS, raw connectivity matrices are utilized, the result is independent of sparsity of the network. The NBS technique has been described in detail by (Zalesky et al. 2010). Here, NBS is used to identify clusters of altered connections specific to narratives containing a particular emotional attachment representation.

NBS was performed in both directions. That is, to identify a subnetwork that in the baseline condition is stronger than all the narrative conditions and vice versa. Furthermore, each of the three narrative conditions was compared separately to the baseline condition. Furthermore, all narrative conditions were also compared with each other. Because of these repeated tests, a Bonferroni correction was applied to the significance level, resulting in a critical level of *α *= 0.0125.

#### Network construction

Based on correlation matrices, weighted, undirected graph networks with 104 nodes were constructed at the individual subject level. The graphs were rendered sparse by recursively removing edges, beginning with the weakest weights and progressing until a certain percentage of edges remained. In the event where removing an edge would disconnect the graph, this edge was retained even in the case of low weight and the next weakest edge was removed instead. In order to investigate the influence of different sparsity threshold levels on network properties, nine sparsity thresholds were tested, starting from 12% in increasing steps of 2% to 28%.

#### Nodal graph metrics

In addition to identifying differences in connectivity patterns using NBS, nodal graph metrics can be used to attribute a specific value to characterize every node in a brain network. These metrics can be used to investigate differences between experimental conditions with regard to the involvement of nodes. The most basic graph metric, the degree of a node, is the number of its adjacent edges. Nodal strength is the sum of weights of edges connected to the node. The nodal clustering coefficient indexes the ratio of triangles around a node and is equivalent to the fraction of node neighbors that are neighbors of each other. Efficient local information transfer can be derived from a high clustering coefficient, as it is a measure of segregation. Local efficiency describes the extent of information transfer of the respective node with all other nodes in the network. LEGE is the local efficiency normalized by the global efficiency. The nodal betweenness centrality index represents the fraction of all shortest paths in the network that contain a given node. Nodes with a high betweenness centrality index participate in a large number of shortest paths. The (“out degree”) participation index is a measure of diversity of intermodular connections of individual nodes. The participation index of a node is low, if the node is embedded within local communities, and it is high for nodes, which serve as connectors between different modules (Rubinov and Sporns 2010). Network metrics were derived using functions from the BCT toolbox (Rubinov and Sporns 2010).

#### Statistics

To investigate effects of repeated narrative presentation on brain network organization, statistical analysis of nodal graph metrics was performed utilizing r software (R Core Team, 2012). A Shapiro‐Wilk test revealed that nodal metrics were not normally distributed and therefore nonparametric tests were therefore conducted. A Friedman rank sum test was calculated to examine the effect of conditions within subjects. Secondly, we tested whether more than two conditions were independent using a Kruskal–Wallis test. Multiple comparisons correction was performed using a false discovery rate (FDR) to correct for the number of tested ROIs using the Benjamini–Hochberg (BH) method (Benjamini and Hochberg, 1995). Post hoc testing was performed to identify differences between conditions for ROIs where FWE‐corrected Kruskal–Wallis tests showed a significant difference between conditions. To test for differences between narratives, each pair of conditions was directly compared using a Wilcoxon Signed Rank test, corrected for multiple comparisons using Bonferroni correction.

## Results

### Network‐based statistics

Using NBS, a subnetwork of connections associated with the changing narrative condition was found when considering the contrast baseline > all narratives. This subnetwork survived FWE corrected *P* < 0.0125 at thresholds 3.4, 3.3, and 3.2 (see Table 1). We observed that by decreasing the threshold parameter, the size of the identified subnetwork grows. For this reason, henceforth we term the smallest subnetwork “core‐subnetwork”.

**Table 1:** Significant results of network‐based (NBS) statistics analysis for the two contrasts baseline > all narratives and baseline > dismissing condition. Primary *t*‐statistic thresholds, resulting *P*‐values of the identified subnetworks, as well as their sizes are listed

Comparison	Threshold	*P*‐value	# Nodes	# Edges
Baseline > all narratives	3.2	0.007	51	73
	3.3	0.006	43	56
	3.4	0.008	30	35
Baseline > dismissing	3.4	0.005	41	73
	3.6	0.005	28	44
	3.8	0.006	17	22
	4	0.008	9	9

No network was found at any threshold for the reversed contrast: all narratives > baseline, showing no consistent increase in functional connectivity after listening to any narrative.

**Figure 2:**
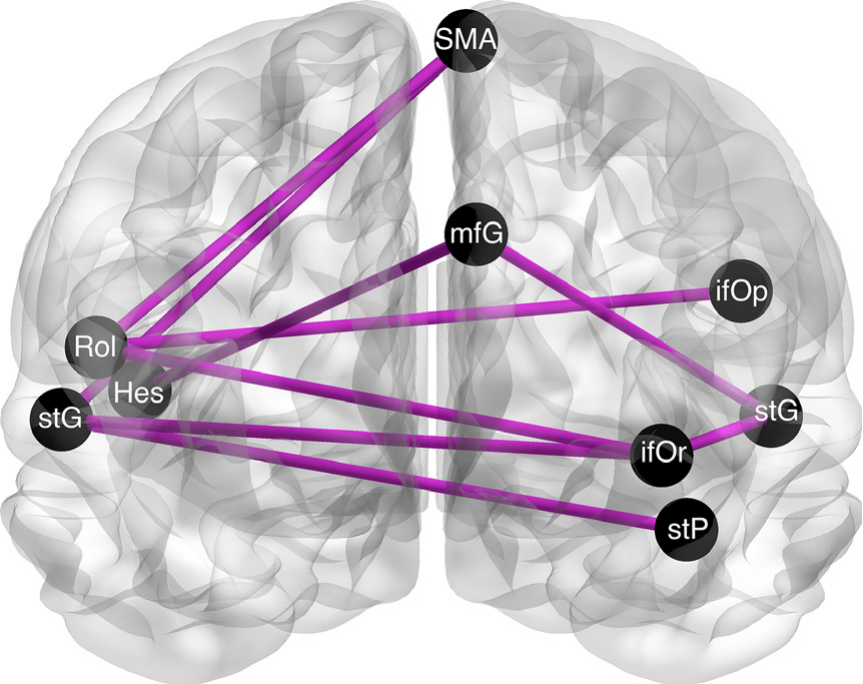
Depiction of the static network‐based statistics “core‐subnetwork” (consisting of 9 nodes and 9 links, threshold = 4) showing reduced connections in the resting phase after listening to the dismissing narrative when compared to the baseline resting condition. See Table 2 for node abbreviations. This visualization was created using BrainNet Viewer Toolbox (Xia et al., 2013).

When considering the contrast baseline > dismissing, another subnetwork of connections was found. Significant (*P* < 0.0125, FWE) subnetworks were found at thresholds 3.4, 3.6, 3.8, and 4.0 (see Table 1). The nine regions involved in this “core‐subnetwork” were: the left supplementary motor area (SMA), both superior temporal lobes, left superior temporal pole, right rolandic operculum, right Heschl's gyrus, orbital part of the left inferior frontal gyrus, left lower superior medial frontal gyrus, and left inferior frontal lobe (see Fig. 2 and Table 2). No network was found for the reversed contrast dismissing > baseline, showing no consistent increase in functional connectivity after listening to the dismissing narrative. No results were found for the remaining contrasts preoccupied/secure narrative versus baseline and vice versa.

**Table 2:** Links of the network‐based statistics (NBS) “core‐subnetwork” (consisting of 9 nodes (AAL regions) and 9 links, threshold = 4) in the contrast baseline > dismissing. Because an undirected network is investigated, here a connection from ROI A to ROI B is symmetrical, implying that the reverse direction is also true

ROI A	ROI B
Left supplementary motor area (SMA)	Right superior temporal gyrus (stG)
Left supplementary motor area (SMA)	Right rolandic operculum (Rol)
Left inferior frontal gyrus, opercular part (ifOp)	Right rolandic operculum (Rol)
Right Heschl gyrus (Hes)	Left lower superior medial frontal gyrus (mfG)
Left superior temporal gyrus (stG)	Left lower superior medial frontal gyrus (mfG)
Left inferior frontal gyrus, orbital part (ifOr)	Right rolandic operculum (Rol)
Left inferior frontal gyrus, orbital part (ifOr)	Left superior temporal gyrus (stG)
Left inferior frontal gyrus, orbital part (ifOr)	Left superior temporal gyrus (stG)
Right superior temporal gyrus (stG)	Left superior temporal pole (stP)

### Nodal graph metrics

**Figure 3:**
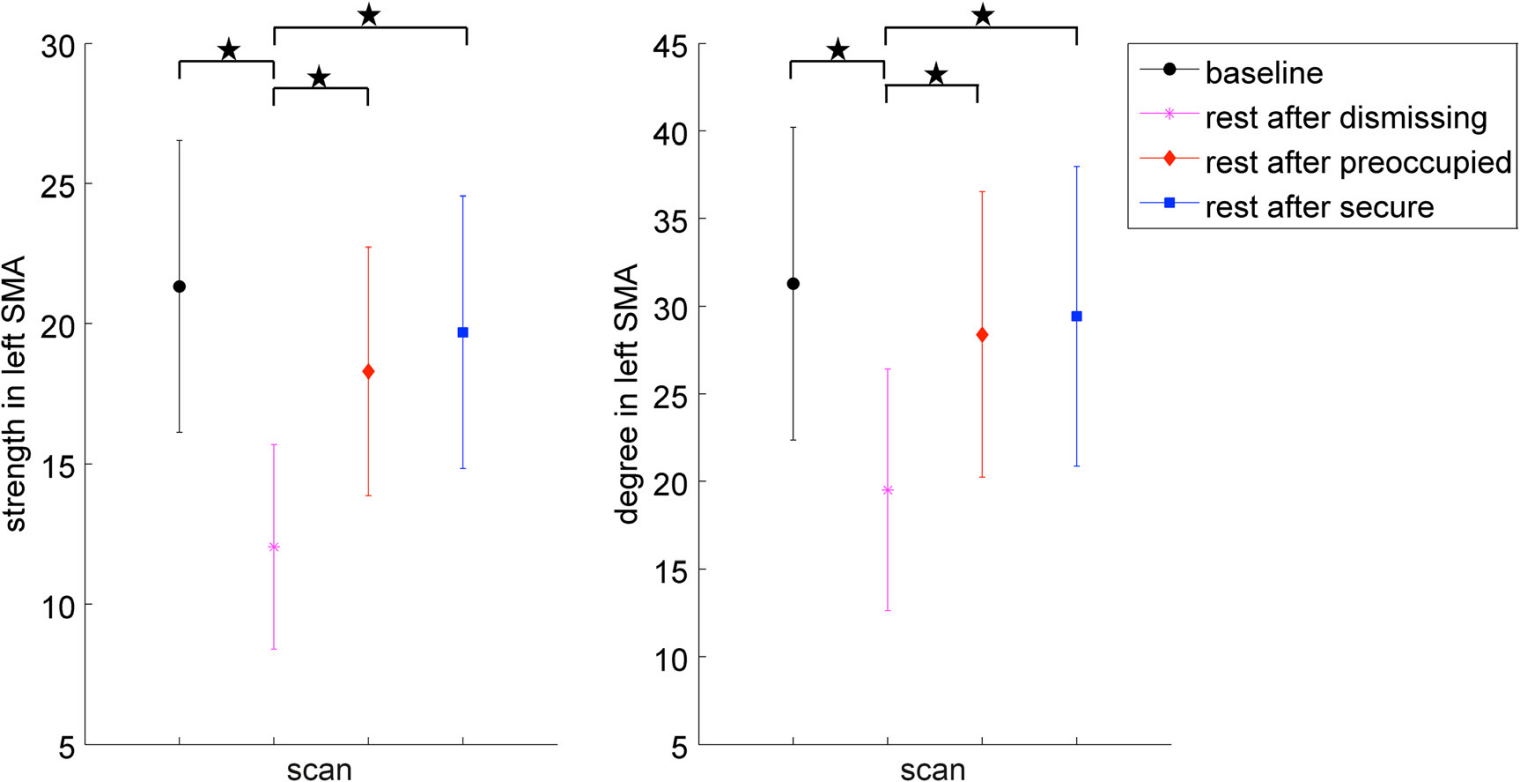
Area‐under‐the curve (AUC) plot showing significant differences (*P* < 0.05, as indicated by black stars) across all tested sparsity thresholds between the dismissing condition and all other conditions in the two local graph metrics strength (left) and degree (right) in the left supplementary motor area (SMA).

Statistical analysis of the local graph metrics with regard to changing narrative condition revealed significant (*P* < 0.05, corrected) differences across all tested sparsity thresholds between listening to the dismissing audiotape compared to the other three conditions (Fig. 3). These differences were restricted to one ROI, the left supplementary motor area (SMA), where decreased overall connectivity of the left SMA in terms of degree and strength was found (Table 3, Table S1).

**Table 3:** List of differences in static local graph metrics after false discovery rate (FDR) corrected results of multiple Mann–Whitney *U* Tests at a significance level of *P* < 0.05. Each difference in degree or strength was only found in the left supplementary motor area regions of interest (SMA ROI). The *P*‐value range indicates maximum and minimum statistical threshold for individual sparsity thresholds. Note that all individual sparsity thresholds revealed significant differences (see also Table S1 for further details)

Metric	Condition	Condition	Sparsity range (%)	*P*‐value range
Strength	Baseline	Dismissing	12–28	0.001–0.004
	Preoccupied	Dismissing	12–28	0.008–0.027
	Secure	Dismissing	12–28	0.001–0.003
Degree	Baseline	Dismissing	12–28	0.001–0.004
	Preoccupied	Dismissing	12–28	0.006–0.032
	Secure	Dismissing	12–28	0.002–0.005

### Connectivity pattern of left supplementary motor area

To scrutinize the finding of a decreased connectivity of the left SMA, the connectivity pattern during the dismissing condition of this specific node was compared with the three remaining conditions.

**Figure 4:**
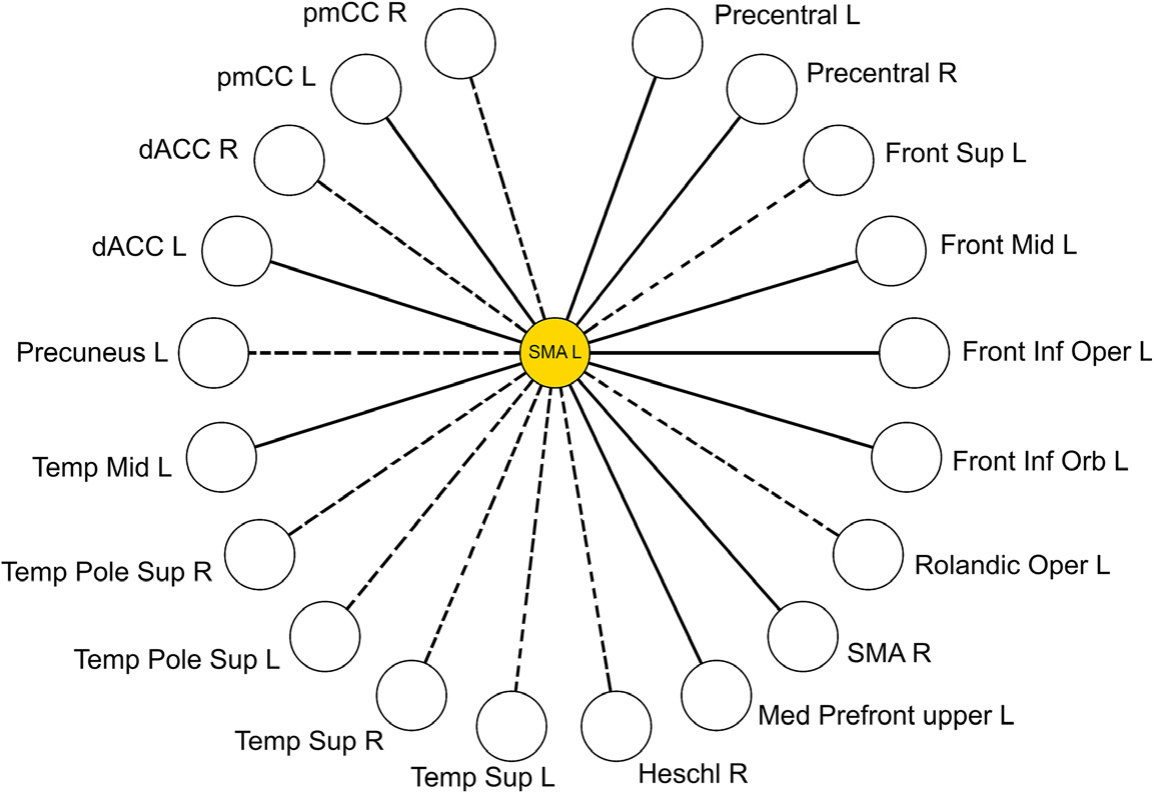
Connectivity pattern of the left supplementary motor area (SMA) (centered yellow node). Each white node depicted in the peripheral circle corresponds to an area the left SMA functionally connects to during the baseline condition. Regions that disconnect from the left SMA in the dismissing condition are depicted with a dashed line.

Comparing the connectivity pattern of the left SMA during the four conditions, the previously described reduction in connections in the dismissing condition was again evident. In the baseline condition, the left SMA was connected to 20 regions. In the preoccupied and secure conditions, the left SMA expressed 16 and 15 links, respectively. However, in the dismissing condition, the number of connecting regions dropped by 50% to 10 links. Regions showing changed connectivity strength to SMA in the dismissing condition, regardless of their inclusion into the NBS “core network”, are visualized in Figure 4. Five temporal regions (both superior temporal gyri, both superior temporal of the temporal pole, right Heschl's gyrus) and 2 frontal (left dorsolateral superior frontal gyrus, left rolandic operculum) as well as the left precuneus, right dorsal anterior cingulate cortex, and right posterior middle cingulate cortex disconnected in the dismissing condition. To explore its uniqueness, we further investigated changes of SMA connectivity pattern in the preoccupied and secure conditions. Overlapping and disparate connections are presented in Table 4.

**Table 4:** Connectivity pattern of left SMA for all 4 conditions at a sparsity threshold of 18%. Only regions the left SMA connects to in the baseline condition in ≥ 13 of 23 subjects are listed together with the percentage of subjects having this connection in their network. For the other conditions, dark table entries represent connections that occurred in ≤ 12 subjects. Please refer to Table S3 for the full list

Left SMA connects to	% of Subjects			
	Baseline	Preoccupied	Secure	Dismissing
Precentral L	100	100	96	92
Precentral R	79	83	61	61
Frontal Inf Oper L	74	61	87	66
Frontal Inf Orb L	79	83	87	70
Supp Motor Area R	100	100	96	100
Temporal Mid L	70	74	92	57
Medial Prefront upper L	74	70	70	74
Dorsal ACC L	79	79	70	61
Posterior MCC L	83	87	70	70
Frontal Sup L	61	79	61	48
Temporal Pole Sup L	74	74	83	44
Posterior MCC R	79	79	66	53
Temporal Sup L	61	66	53	22
Temporal Sup R	66	61	53	22
Dorsal ACC R	57	61	48	35
Temporal Pole Sup R	57	53	61	31
Frontal Mid L	57	44	57	70
Heschl R	57	44	53	18
Rolandic Oper L	57	44	48	22
Precuneus L	57	27	44	18

SMA, supplementary motor area.

### Dynamic local graph metrics

To investigate dynamic characteristics of SMA connectivity between the narrative conditions, we split the rs‐fMRI dataset into three nonoverlapping time‐intervals of equal length, each containing 76 volumes, corresponding to 3.2 min per time interval. Subsequently, connectivity matrices were constructed for each interval in the same way as described above. For post hoc analysis of dynamic local graph metrics, only degree and strength of the left SMA were computed at a sparsity threshold of 18%. These values were normally distributed.

For ease of interpretation, we normalized graph metric values to baseline level by subtraction of the baseline condition group mean value for each time‐interval from each subject.

Repeated‐measures *t*‐tests between all narrative conditions in similar time intervals were computed, and FDR correction was applied.

**Figure 5:**
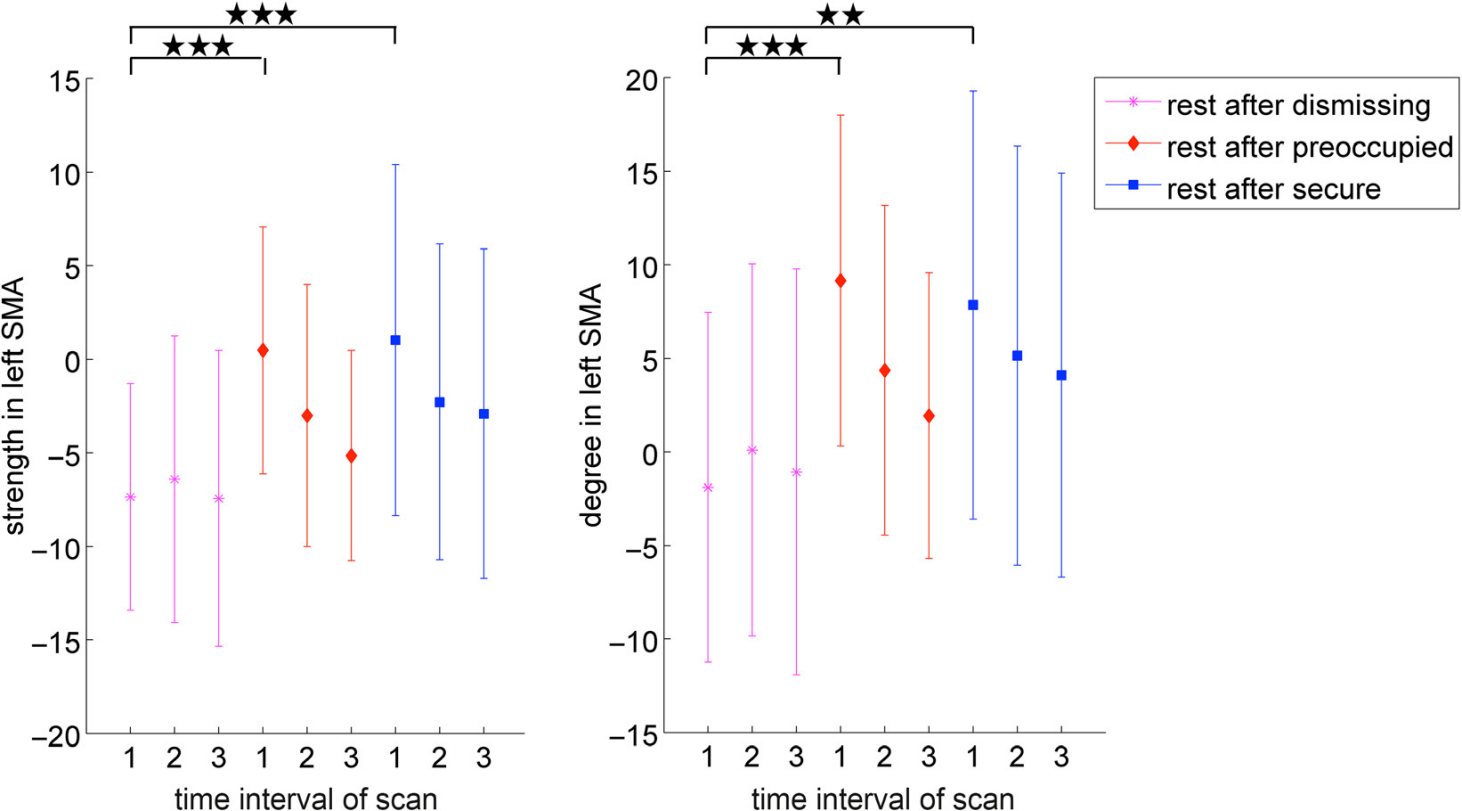
False discovery rate corrected differences in the two local graph metrics strength (left) and degree (right) of the left supplementary motor area at a sparsity threshold of 18% between time intervals after normalization to baseline condition. ***: *P* < 0.001 **: *P* < 0.01.

During the first time interval, both degree and strength of the left SMA were significantly decreased in the dismissing condition when compared to both preoccupied (for degree *P* = 3.5 × 10^−4^ and for strength *P* = 3.3 × 10^−4^) and secure conditions (for degree *P* = 2.9 × 10^−3^ and for strength *P* = 8.8 × 10^−4^) (Fig. 5).

This difference dissolved over the duration of the resting‐state measurement. There was no evidence for a difference between preoccupied and secure conditions in any of the time intervals.

### Mood and correlates of personality

To assess whether the experiment influenced mood, vigilance, and agitation of the participants, a paired *t*‐test was performed to compare subscales of the MDBF questionnaire before and after the experiment. No significant differences showed that subjects' mood (*P* = 0.47), vigilance (*P* = 0.12), and agitation (*P* = 0.19) were unaffected.

To assess whether subclinical depression, maltreatment experiences, or personality dimensions correlate with connectivity of the left SMA, correlation coefficients between BDI score, the CTQ total score and its six subscales, as well as the seven subscales of the TCI with the degree of the left SMA were calculated for both the baseline condition and the dismissing condition. Because none of the correlation coefficients were significant (all *P*‐values > 0.05), an influence of emotional symptoms on baseline and on the aberrant network characteristics in the dismissing condition is excluded.

A correlation between the degree of SMA in both baseline and dismissing conditions and MDBF scores for mood, vigilance and agitation measured before the experiment was not significant (all *P*‐values > 0.05) showing that prior emotional state does not correlate with the connectivity pattern of the left SMA.

## Discussion

Graph‐theory based connectivity analyses of resting‐state fMRI scans acquired after participants listened to emotional biographic narratives (post‐task rs‐fMRI), identified a brain network, including the left SMA, specifically representing decreased connectivity after a dismissing narrative when compared with a baseline resting condition, indicating profound differences in ICN patterns regarding emotional reactivity between conditions.

In the static time series analysis, decreases in the two graph metrics, degree and strength of the left SMA, characterized differences between baseline and dismissing conditions, which suggests a crucial role of the left SMA in emotional reactivity along with impacted processing of stimuli with negative valence. The left SMA is the only area exhibiting a reduction in degree and strength when functional connections to temporal and frontal regions are removed. In an additional dynamic approach, these changes in ICNs between the dismissing and both the preoccupied and the secure condition were shown to be strongest in the first time interval.

### Convergence of NBS and connectivity pattern analysis

The surprisingly sparse, though tightly focused results in local graph metrics motivated a subsequent analysis of the connectivity patterns of the left SMA during the four conditions. While the local metrics degree and strength detail the number of connections of an area and their strength, respectively, the connectivity pattern could reveal which links are retained or lost among the different conditions.

With the aim to draw comparisons between obtained findings, we compared the “core‐subnetwork” resulting from NBS analyses against the change in connectivity patterns of the left SMA and found an overlap. The NBS “core‐subnetwork” contains nine nodes (amongst others the left SMA), out of which six connect to the left SMA in the baseline condition. Four of these six ROIs (both superior temporal gyri, right Heschl's gyrus, and left superior temporal gyrus of the temporal pole) showed a reduced connectivity pattern in the dismissing condition compared to the baseline condition. The other two overlapping ROIs are: the right rolandic operculum (the left rolandic operculum appears in the SMA Connectivity Pattern), and the left lower medial superior frontal gyrus (the left upper medial superior frontal gyrus appears in the SMA connectivity pattern). This overlap of 35% is highly above chance. Chances for picking seven out of 20 from 104 ROIs without replacement by random chance are: 6.33 × 10^−12^. However, one has to keep in mind that only the most consistent connections at the group level were investigated for the left SMA. Therefore, it is possible that the reduced connectivity speaks more toward a less consistent pattern at the group level rather than less connected pattern. However, the reduced degree of the left SMA, coupled with its inclusion in the NBS result, shows both a reduction in the number of connections and that the individual correlations are reduced.

### Interpretations

Changes in the mental state of the listener are not solely evoked by the external auditory input but rather an interaction of the respective conveyed emotional state of the interviewee and the listener's individual internal disposition to engage in social interactions. Activation of a network, in which the left SMA is a major player, may reflect changes associated with emotional reactivity from the biographical narrative to the listener. Our daring interpretation is that emotional entanglement with the content of the narrative can be seen as a form of individual interpersonal reactivity, such as frequently observed during countertransference mechanisms. Countertransferance is a psychoanalytical concept of interpersonal reactivity, describing the unknowing unconsciously elicited redirection of own experience of emotional states in a listener.

Moreover, this study produced evidence that further supports the idea of carry‐over effects following a cognitively demanding task studied by (Barnes et al. 2009). The characteristic change in ICN pattern in the dismissing condition confirms the influence of valence of exogenous stimulation on endogenous dynamics and indicates once more that changes of the mental state lead to changes of brain connectivity.

#### Role of the narratives

A prior study by Kirchmann et al. found that a preoccupied narrative evoked the highest impairment of well‐being, as assessed by a questionnaire given to the subjects after listening to the narrative (Kirchmann et al. 2011).

An investigation by Morriss et al. measured the time course of emotional recovery with electroencephalography, and their results demonstrate that emotional events modulate subsequent processing and, more specifically, that negative stimuli impact upon subsequent processing for a longer duration than positive stimuli do (Morriss et al. 2013). Regarding results of this study, it is certain that the changes after listening to the dismissing narrative are particularly intense.

Concerning the absence of significant alterations in connectivity patterns of ICN in the secure and preoccupied conditions, we can only speculate that these narratives do not influence or disturb the listeners to the same extent as the dismissing tape does. In this way, the content of the dismissing narrative might have evoked a need for increased cognitive processing or increased efforts to maintain well‐being as opposed to the others (Van Harmelen et al. 2013, 2014).

We interpret the decrease in degree of the left SMA after the dismissing narrative as evidence for a centralized and eminent effect regarding organization of the ICN as a whole.

Despite the fact that the exact neuronal mechanisms involved in effects of attachment on emotional processing and regulation remain largely unexplored, evidence from recent neuroimaging studies suggests a stronger recruitment of neural systems active during negative emotional states during processing of attachment‐related information, and impaired regulatory capacities to inhibit such processing (Mikulincer and Shaver 2007; Vrticka and Vuilleumier 2012).

However, behavioral studies revealed that individual differences in attachment representation correlate with differences in several cognitive and affective processes, particularly in attachment‐relevant or social contexts (for example Rognoni et al. 2008; Vrticka and Vuilleumier 2012). Mikulincer et al. were able to show that an ambivalent attachment representation leads to heightened processing of attachment‐related information in general, whereas a dismissing attachment representation entails specific opposite effects during negative contexts (Mikulincer et al. 2002).

Evidence for a aberrant ICN configuration in the dismissing condition, which includes the strongest negative social cues, could accordingly be explained if the majority of the participants in this study have an insecure (dismissing or preoccupied) attachment representation themselves.

Another possible interpretation of the disconnected ICN pattern in the dismissing condition might be a disengagement from the stimulus material. Interestingly, this disengagement mirrors experiences that dismissing individuals have had with their caregivers in the past. This interpretation is corroborated by evidence from Krause et al., who found that participants showed significant differences regarding the tendency to engage in potential social interaction with the narrative characters, the smallest tendency being reported for the dismissing condition (Krause et al., in revision).

Therefore, it might be possible that as a consequence of the countertransference reaction, listeners disregard the dismissing individuals.

#### Role of the SMA

Due to its direct and substantial connections to the cortico‐spinal tract as well as activity before movements occur, the role of the SMA has again and again been attributed to preparation of motor imagery and output (Kornhuber and Deecke 1965; Tanji and Kurata 1982; Halder et al. 2011). Furthermore, connections of the SMA to the basal ganglia are thought to have the potential to rapidly interfere with cortical‐basal ganglia circuits (Nachev et al. 2008).

Fan et al. performed a whole‐brain‐based quantitative meta‐analysis of fMRI studies of empathy and identified a “core network of empathy”, consisting of dorsal anterior cingulate cortex (dACC), anterior medial cingulate cortex (aMCC), SMA, and bilateral insula (Fan et al. 2011). The right SMA was identified as being active in the affective‐perceptual form of empathy.

More specifically, the SMA has been implicated in imagination of pain caused to oneself or to others in normal participants (Decety et al. 2013b). Moreover, the left SMA in particular was found to be involved in empathic responses involving anger (De Greck et al. 2012). Participants with a diagnosis of psychopathy, deemed to have diminished empathy, showed similar SMA activation to normal participants when imagining pain caused to themselves, but this activation was not present when asked to imagine pain caused to others (Decety et al. 2013a). Patients with schizophrenia have also been found to show reduced empathy (Bora et al. 2008; Lee et al. 2011) and a trend to a reduction in SMA activity has been shown in patients with schizophrenia during a motor task (Schröder et al. 1995). Furthermore, changes in SMA gray matter volume have been identified in patients with schizophrenia with motor abnormalities (Stegmayer et al. 2014). The present findings are consistent with a role for the SMA in empathy, and in particular that involving pain and anger, which are relevant to the dismissing narrative.

Most likely, the SMA shows a functional pleomorphism by overtaking different functions in different circumstances (Nachev et al. 2008).

A potential explanation for involvement of the SMA might be that because in the dismissing narrative the discomforting emotional state of the interviewee is redirected towards the listener and this countertransference reaction elicits a unique impulse to act in the listener. Such an emotional reaction might then be converted into a not executed, but internalized motor response.

Such an interpretation is in line with a theory by Goldberg, which states that the SMA is primarily involved in the control of movements triggered internally, while external stimuli (e.g., visual‐spatial, auditory, proprioceptive) trigger activation of the premotor area which is primarily involved in control of movements (Goldberg 1985). In 2003, Oliveri et al. stimulated the SMA with transcranial magnetic stimulation (TMS) during emotional and nonemotional visually cued movements and concluded that the SMA could interface the limbic and motor systems in the transformation of emotional experiences into motor actions (Oliveri et al. 2003). Another study by Rodigari et al. confirmed this hypothesis and described the SMA as a core node in the brain network for emotional control (Rodigari and Oliveri 2014). Following repetitive TMS to the SMA, perceived valence of negative emotions was significantly increased, which is compatible with the existence of an inhibitory connectivity between SMA and regions of the limbic system involved in processing negative emotions suggesting a role for the SMA in top‐down inhibitory control of conflicting responses, whether emotional or purely motor.

### Limitations

Properties of graph networks also depend on the chosen parcellation scheme and preprocessing steps (Bassett et al. 2006; Zalesky et al. 2010; Braun et al. 2012; Liang et al. 2012). For this reason, the current results may be restricted to the applied setting parameters. As there is no established rule for choosing an appropriate network density, we examined a wide range of sparsity thresholds and found significant evidence for altered SMA connectivity across the entire range tested (see Table 3).

For analysis of the connectivity pattern of the SMA, we chose a sparsity threshold of 18%, because it would be a medial value considering the overall range of tested sparsities and this threshold not only represents a robust choice, but is also in line with previous literature reports (Borchardt et al. 2014). To increase specificity of the observations, a connection between the left SMA and another ROI was used if it occurred in the networks of at least 13 of 23 subjects. Differences between absence and presence of connections of the left SMA were not tested for significance. However, the full list of connections of the left SMA is provided in Table S3.

Regarding the dynamic graph analysis, one might question the adequacy of the length of the time intervals, as they might not accurately sample the recovery or carry‐over effect. We do not expect the timescale of recovery to be shorter than three minutes, because in this case the effect seen in the static analysis would have vanished. This was not the case in the current dataset. However, the timescale of recovery could possibly be longer than three minutes, which would require an investigation in greater detail, in which the use of a sliding‐window approach could be taken into consideration.

### Future work

The differential influence of a listener's own attachment representation on emotional reactivity is an interesting aspect that is worth to be investigating in future research. This is of paramount interest, considering therapy conversations between patients and mental health professionals regarding engagement of the practitioner with the patient's narrative and the subsequent unconscious redirection of the practitioner's feelings towards the patient (Muller 2009). Investigation of such emotional reactivity in patients suffering from conditions known to affect empathy, such as affective disorders, schizophrenia, and psychopathy could potentially shed new light on the mechanisms underlying reduced empathy, leading to specific diagnostic and treatment approaches. Furthermore, dynamic effects of changing mental states could be investigated at a higher temporal resolution by means of simultaneous EEG‐fMRI, which would be of tremendous interest.

## Conclusion

In this post‐task resting‐state fMRI study, we identified changes in intrinsic functional connectivity networks evoked by exogenous auditory stimuli with high social affective content. After having listened to a biographical narrative, decreases in the connectivity strength of a brain network consisting of the left SMA, both superior temporal lobes, left superior temporal pole, right rolandic operculum, right Heschl's gyrus, orbital part of the left inferior frontal gyrus, left lower superior medial frontal gyrus, and left inferior frontal lobe are interpreted as dynamic switching between processing stages in the brain in reaction to the emotional content of the audiotape.

Results of the dynamic time series analysis emphasize that the observed effects in the static whole time series emerge from brain state changes occurring during the initial three minutes of the resting phase after exogenous auditory stimulation.

Evidence from this study clearly supports the relevance of the left SMA regarding emotional reactivity to stimuli with negative valence.

The interpersonal reactivity is interpreted as a form of countertransference, where changes in mood of the listener are a result of external and internal circumstances streaming in the consciousness of the listener.

This study provides additional evidence with respect to the carry‐over effects following an emotional task, and indicates once more that changes of the mental state lead to subsequent changes in brain connectivity. The extent to which such long‐term state changes appear, however, seems crucially dependent on the type of social information and the individual behavioral relevance.

## Conflict of interest

None declared.

## Supporting information


**Table S1.** Complete table of significant differences (*P* < 0.05, FDR corrected) in nodal graph metrics between all four conditions.
**Table S2.** Abbreviated names of 105 regions of interest, their MNI coordinates, and class of brain region. L and R stand for left and right, respectively.
**Table S3.** Full connectivity pattern of left SMA at a sparsity threshold of 18%. Percentage of subjects having a connection to the left SMA is listed for every of the four experimental conditions. Columns printed in bold are also listed in Table 4.

**Figure S1.** Insula and cingulate cortex parcellation in neurological orientation. (A) Bilateral anterior and posterior insula ROIs are shown in red and blue, respectively. (B) Unilateral cingulate subregions: rostral ACC (cyan), pregenual ACC (yellow), dorsal ACC (blue), posterior MCC (purple), PCC (BA23, red), dorsal PCC (blue), ventral PCC (green).
