# Supplementary material for: Dynamic disconnection of the supplementary motor area after processing of dismissive biographic narratives
Source: Brain Behav. 2015 Sep 14;5(10):e00377. doi: 10.1002/brb3.377 (PMC4614061; doi:10.1002/brb3.377)
Supplement: Supplementary file 1 — Table S1. Complete table of significant differences (P < 0.05, FDR corrected) in nodal graph metrics between all four conditions. Table S2. Abbreviated names of 105 regions of interest, their MNI coordinates, and class of brain region. L and R stand for left and right, respectively. Table S3. Full connectivity pattern of left SMA at a sparsity threshold of 18%. Percentage of subjects having a connection to the left SMA is listed for every of the four experimental conditions. Columns printed in bold are also listed in Table 4. [file BRB3-5-0i-s001.docx]

**Supplementary Material**

**Table S1:** Complete table of significant differences (p<0.05, FDR corrected) in nodal graph metrics between all 4 conditions.

| **sparsity** | **metric** | **p-value** | **condition** | **condition** |
| --- | --- | --- | --- | --- |
| 12 | Strength | 0.0029 | baseline | dismissing |
| 12 | Strength | 0.0098 | preoccupied | dismissing |
| 12 | Strength | 0.0026 | secure | dismissing |
| 12 | Degree | 0.0041 | baseline | dismissing |
| 12 | Degree | 0.0057 | preoccupied | dismissing |
| 12 | Degree | 0.0034 | secure | dismissing |
| 14 | Strength | 0.0010 | baseline | dismissing |
| 14 | Strength | 0.0121 | preoccupied | dismissing |
| 14 | Strength | 0.0029 | secure | dismissing |
| 14 | Degree | 0.0013 | baseline | dismissing |
| 14 | Degree | 0.0112 | preoccupied | dismissing |
| 14 | Degree | 0.0043 | secure | dismissing |
| 16 | Strength | 0.0041 | baseline | dismissing |
| 16 | Strength | 0.0109 | preoccupied | dismissing |
| 16 | Strength | 0.0033 | secure | dismissing |
| 16 | Degree | 0.0034 | baseline | dismissing |
| 16 | Degree | 0.0121 | preoccupied | dismissing |
| 16 | Degree | 0.0051 | secure | dismissing |
| 18 | Strength | 0.0023 | baseline | dismissing |
| 18 | Strength | 0.0079 | preoccupied | dismissing |
| 18 | Strength | 0.0016 | secure | dismissing |
| 18 | Degree | 0.0023 | baseline | dismissing |
| 18 | Degree | 0.0075 | preoccupied | dismissing |
| 18 | Degree | 0.0016 | secure | dismissing |
| 20 | Strength | 0.0026 | baseline | dismissing |
| 20 | Strength | 0.0098 | preoccupied | dismissing |
| 20 | Strength | 0.0016 | secure | dismissing |
| 20 | Degree | 0.0023 | baseline | dismissing |
| 20 | Degree | 0.0082 | preoccupied | dismissing |
| 20 | Degree | 0.0019 | secure | dismissing |
| 22 | Strength | 0.0026 | baseline | dismissing |
| 22 | Strength | 0.0109 | preoccupied | dismissing |
| 22 | Strength | 0.0014 | secure | dismissing |
| 22 | Degree | 0.0025 | baseline | dismissing |
| 22 | Degree | 0.0108 | preoccupied | dismissing |
| 22 | Degree | 0.0019 | secure | dismissing |
| 24 | Strength | 0.0029 | baseline | dismissing |
| 24 | Strength | 0.0164 | preoccupied | dismissing |
| 24 | Strength | 0.0016 | secure | dismissing |
| 24 | Degree | 0.0023 | baseline | dismissing |
| 24 | Degree | 0.0177 | preoccupied | dismissing |
| 24 | Degree | 0.0026 | secure | dismissing |
| 26 | Strength | 0.0037 | baseline | dismissing |
| 26 | Strength | 0.0220 | preoccupied | dismissing |
| 26 | Strength | 0.0026 | secure | dismissing |
| 26 | Degree | 0.0043 | baseline | dismissing |
| 26 | Degree | 0.0296 | preoccupied | dismissing |
| 26 | Degree | 0.0037 | secure | dismissing |
| 28 | Strength | 0.0037 | baseline | dismissing |
| 28 | Strength | 0.0267 | preoccupied | dismissing |
| 28 | Strength | 0.0016 | secure | dismissing |
| 28 | Degree | 0.0035 | baseline | dismissing |
| 28 | Degree | 0.0322 | preoccupied | dismissing |
| 28 | Degree | 0.0031 | secure | dismissing |

**Table S2:** Abbreviated names of 105 regions of interest, their MNI coordinates, and class of brain region. L and R stand for left and right, respectively.

| **name** | **x** | **y** | **z** | **class** |
| --- | --- | --- | --- | --- |
| Precentral L | -38.65 | -5.68 | 50.94 | Primary |
| Precentral R | 41.37 | -8.21 | 52.09 | Primary |
| Frontal Sup L | -18.45 | 34.81 | 42.2 | Association |
| Frontal Sup R | 21.9 | 31.12 | 43.82 | Association |
| Frontal Sup Orb L | -16.56 | 47.32 | -13.31 | Paralimbic |
| Frontal Sup Orb R | 18.49 | 48.1 | -14.02 | Paralimbic |
| Frontal Mid L | -33.43 | 32.73 | 35.46 | Association |
| Frontal Mid R | 37.59 | 33.06 | 34.04 | Association |
| Frontal Mid Orb L | -30.65 | 50.43 | -9.62 | Paralimbic |
| Frontal Mid Orb R | 33.18 | 52.59 | -10.73 | Paralimbic |
| Frontal Inf Oper L | -48.43 | 12.73 | 19.02 | Association |
| Frontal Inf Oper R | 50.2 | 14.98 | 21.41 | Association |
| Frontal Inf Tri L | -45.58 | 29.91 | 13.99 | Association |
| Frontal Inf Tri R | 50.33 | 30.16 | 14.17 | Association |
| Frontal Inf Orb L | -35.98 | 30.71 | -12.11 | Paralimbic |
| Frontal Inf Orb R | 41.22 | 32.23 | -11.91 | Paralimbic |
| Rolandic Oper L | -47.16 | -8.48 | 13.95 | Association |
| Rolandic Oper R | 52.65 | -6.25 | 14.63 | Association |
| Supp Motor Area L | -5.32 | 4.85 | 61.38 | Association |
| Supp Motor Area R | 8.62 | 0.17 | 61.85 | Association |
| Olfactory L | -8.06 | 15.05 | -11.46 | Primary |
| Olfactory R | 10.43 | 15.91 | -11.26 | Primary |
| Frontal Med Orb L | -5.17 | 54.06 | -7.4 | Association |
| Frontal Med Orb R | 8.16 | 51.67 | -7.13 | Association |
| Rectus L | -5.08 | 37.07 | -18.14 | Paralimbic |
| Rectus R | 8.35 | 35.64 | -18.04 | Paralimbic |
| Hippocampus L | -25.03 | -20.74 | -10.13 | Limbic |
| Hippocampus R | 29.23 | -19.78 | -10.33 | Limbic |
| ParaHippocampal L | -21.17 | -15.95 | -20.7 | Paralimbic |
| ParaHippocampal R | 25.38 | -15.15 | -20.47 | Paralimbic |
| Amygdala L | -23.27 | -0.67 | -17.14 | Limbic |
| Amygdala R | 27.32 | 0.64 | -17.5 | Limbic |
| Calcarine L | -7.14 | -78.67 | 6.44 | Primary |
| Calcarine R | 15.99 | -73.15 | 9.4 | Primary |
| Cuneus L | -5.93 | -80.13 | 27.22 | Association |
| Cuneus R | 13.51 | -79.36 | 28.23 | Association |
| Lingual L | -14.62 | -67.56 | -4.63 | Association |
| Lingual R | 16.29 | -66.93 | -3.87 | Association |
| Occipital Sup L | -16.54 | -84.26 | 28.17 | Association |
| Occipital Sup R | 24.29 | -80.85 | 30.59 | Association |
| Occipital Mid L | -32.39 | -80.73 | 16.11 | Association |
| Occipital Mid R | 37.39 | -79.7 | 19.42 | Association |
| Occipital Inf L | -36.36 | -78.29 | -7.84 | Association |
| Occipital Inf R | 38.16 | -81.99 | -7.61 | Association |
| Fusiform L | -31.16 | -40.3 | -20.23 | Association |
| Fusiform R | 33.97 | -39.1 | -20.18 | Association |
| Postcentral L | -42.46 | -22.63 | 48.92 | Primary |
| Postcentral R | 41.43 | -25.49 | 52.55 | Primary |
| Parietal Sup L | -23.45 | -59.56 | 58.96 | Association |
| Parietal Sup R | 26.11 | -59.18 | 62.06 | Association |
| Parietal Inf L | -42.8 | -45.82 | 46.74 | Association |
| Parietal Inf R | 46.46 | -46.29 | 49.54 | Association |
| SupraMarginal L | -55.79 | -33.64 | 30.45 | Association |
| SupraMarginal R | 57.61 | -31.5 | 34.48 | Association |
| Angular L | -44.14 | -60.82 | 35.59 | Association |
| Angular R | 45.51 | -59.98 | 38.63 | Association |
| Precuneus L | -7.24 | -56.07 | 48.01 | Association |
| Precuneus R | 9.98 | -56.05 | 43.77 | Association |
| Paracentral Lobule L | -7.63 | -25.36 | 70.07 | Association |
| Paracentral Lobule R | 7.48 | -31.59 | 68.09 | Association |
| Caudate L | -11.46 | 11 | 9.24 | Subcortical |
| Caudate R | 14.84 | 12.07 | 9.42 | Subcortical |
| Putamen L | -23.91 | 3.86 | 2.4 | Subcortical |
| Putamen R | 27.78 | 4.91 | 2.46 | Subcortical |
| Pallidum L | -17.75 | -0.03 | 0.21 | Subcortical |
| Pallidum R | 21.2 | 0.18 | 0.23 | Subcortical |
| Thalamus L | -10.85 | -17.56 | 7.98 | Subcortical |
| Thalamus R | 13 | -17.55 | 8.09 | Subcortical |
| Heschl L | -41.99 | -18.88 | 9.98 | Primary |
| Heschl R | 45.86 | -17.15 | 10.41 | Primary |
| Temporal Sup L | -53.16 | -20.68 | 7.13 | Association |
| Temporal Sup R | 58.15 | -21.78 | 6.8 | Association |
| Temporal Pole Sup L | -39.88 | 15.14 | -20.18 | Paralimbic |
| Temporal Pole Sup R | 48.25 | 14.75 | -16.86 | Paralimbic |
| Temporal Mid L | -55.52 | -33.8 | -2.2 | Association |
| Temporal Mid R | 57.47 | -37.23 | -1.47 | Association |
| Temporal Pole Mid L | -36.32 | 14.59 | -34.08 | Paralimbic |
| Temporal Pole Mid R | 44.22 | 14.55 | -32.23 | Paralimbic |
| Temporal Inf L | -49.77 | -28.05 | -23.17 | Association |
| Temporal Inf R | 53.69 | -31.07 | -22.32 | Association |
| Medial Prefront lower L | -6.9 | 58 | 16.9 | Association |
| Medial Prefront lower R | 8.4 | 60.6 | 15.3 | Association |
| Medial Prefront upper L | -4.8 | 40.6 | 44.9 | Association |
| Medial Prefront upper R | 7.6 | 41.1 | 46.4 | Association |
| Ant Insula L | -34.9 | 16.2 | 0.3 | Paralimbic |
| Ant Insula R | 37.3 | 16.7 | -1.7 | Paralimbic |
| Post Insula L | -37.9 | -8.2 | 8.2 | Paralimbic |
| Post Insula R | 38.9 | -8.1 | 7.2 | Paralimbic |
| Rostral ACC L | -4.81 | 31.3 | -6.22 | Paralimbic |
| Rostral ACC R | 2.98 | 30.4 | -5.05 | Paralimbic |
| Pregenual ACC L | -5.55 | 43.8 | 6.72 | Paralimbic |
| Pregenual ACC R | 6.42 | 43 | 9 | Paralimbic |
| Dorsal ACC L | -4.81 | 24.9 | 28.7 | Paralimbic |
| Dorsal ACC R | 6.37 | 28.3 | 29.6 | Paralimbic |
| Posterior MCC L | -5.7 | -8.81 | 40.8 | Paralimbic |
| Posterior MCC R | 5.85 | -9 | 40.9 | Paralimbic |
| PCC BA23 L | -6.68 | -35.4 | 43.3 | Paralimbic |
| PCC BA23 R | 6.26 | -34.6 | 41.6 | Paralimbic |
| dPCC L | -4.98 | -43.8 | 27.4 | Paralimbic |
| dPCC R | 5.05 | -42.5 | 26.5 | Paralimbic |
| vPCC L | -10.9 | -48.1 | 12.1 | Paralimbic |
| vPCC R | 10.9 | -45.7 | 13.4 | Paralimbic |
| TPJ L | -54 | -54 | 28 | Association |
| TPJ R | 54 | -54 | 28 | Association |

**Table S3:** Full connectivity pattern of left SMA at a sparsity threshold of 18%. Percentage of subjects having a connection to the left SMA is listed for every of the 4 experimental conditions. Columns printed in bold are also listed in Table 4.

| left SMA connects to | % of subjects | | | |
| --- | --- | --- | --- | --- |
|  | baseline | preoccupied | secure | dismissing |
| Precentral L | 100 | 100 | 96 | 92 |
| Precentral R | 79 | 83 | 61 | 61 |
| Frontal Inf Oper L | 74 | 61 | 87 | 66 |
| Frontal Inf Orb L | 79 | 83 | 87 | 70 |
| Supp Motor Area R | 100 | 100 | 96 | 100 |
| Temporal Mid L | 70 | 74 | 92 | 57 |
| Medial Prefront upper L | 74 | 70 | 70 | 74 |
| Dorsal ACC L | 79 | 79 | 70 | 61 |
| Posterior MCC L | 83 | 87 | 70 | 70 |
| Frontal Sup L | 61 | 79 | 61 | 48 |
| Temporal Pole Sup L | 74 | 74 | 83 | 44 |
| Posterior MCC R | 79 | 79 | 66 | 53 |
| Temporal Sup L | 61 | 66 | 53 | 22 |
| Temporal Sup R | 66 | 61 | 53 | 22 |
| Dorsal ACC R | 57 | 61 | 48 | 35 |
| Temporal Pole Sup R | 57 | 53 | 61 | 31 |
| Frontal Mid L | 57 | 44 | 57 | 70 |
| Heschl R | 57 | 44 | 53 | 18 |
| Rolandic Oper L | 57 | 44 | 48 | 22 |
| Precuneus L | 57 | 27 | 44 | 18 |

| **ROI** | **baseline** | **preoccupied** | **secure** | **dismissing** |
| --- | --- | --- | --- | --- |
| **Precentral L** | **100** | **100** | **96** | **92** |
| **Precentral R** | **79** | **83** | **61** | **61** |
| **Frontal Sup L** | **61** | **79** | **61** | **48** |
| Frontal Sup R | 53 | 40 | 44 | 27 |
| Frontal Sup Orb L | 27 | 22 | 9 | 9 |
| Frontal Sup Orb R | 9 | 0 | 5 | 0 |
| **Frontal Mid L** | **57** | **44** | **57** | **70** |
| Frontal Mid R | 18 | 14 | 0 | 9 |
| Frontal Mid Orb L | 5 | 18 | 18 | 5 |
| Frontal Mid Orb R | 5 | 0 | 0 | 0 |
| **Frontal Inf Oper L** | **74** | **61** | **87** | **66** |
| Frontal Inf Oper R | 31 | 27 | 22 | 18 |
| Frontal Inf Tri L | 35 | 31 | 48 | 27 |
| Frontal Inf Tri R | 22 | 9 | 18 | 5 |
| **Frontal Inf Orb L** | **79** | **83** | **87** | **70** |
| Frontal Inf Orb R | 31 | 40 | 40 | 22 |
| **Rolandic Oper L** | **57** | **44** | **48** | **22** |
| Rolandic Oper R | 53 | 48 | 31 | 9 |
| Supp Motor Area L | **-** | **-** | **-** | **-** |
| **Supp Motor Area R** | **100** | **100** | **96** | **100** |
| Olfactory L | 0 | 0 | 0 | 0 |
| Olfactory R | 0 | 0 | 0 | 0 |
| Frontal Med Orb L | 14 | 14 | 9 | 9 |
| Frontal Med Orb R | 14 | 5 | 5 | 0 |
| Rectus L | 14 | 5 | 5 | 9 |
| Rectus R | 18 | 0 | 5 | 5 |
| Hippocampus L | 0 | 0 | 0 | 0 |
| Hippocampus R | 0 | 0 | 0 | 0 |
| ParaHippocampal L | 5 | 0 | 0 | 5 |
| ParaHippocampal R | 5 | 0 | 0 | 0 |
| Amygdala L | 0 | 0 | 0 | 0 |
| Amygdala R | 0 | 0 | 0 | 5 |
| Calcarine L | 31 | 5 | 27 | 9 |
| Calcarine R | 18 | 0 | 18 | 9 |
| Cuneus L | 18 | 5 | 5 | 0 |
| Cuneus R | 22 | 9 | 0 | 0 |
| Lingual L | 18 | 9 | 31 | 5 |
| Lingual R | 22 | 5 | 18 | 5 |
| Occipital Sup L | 18 | 14 | 0 | 0 |
| Occipital Sup R | 18 | 9 | 0 | 0 |
| Occipital Mid L | 14 | 18 | 14 | 0 |
| Occipital Mid R | 5 | 14 | 5 | 0 |
| OccipitalInfL | 9 | 14 | 14 | 0 |
| OccipitalInfR | 9 | 18 | 14 | 9 |
| Fusiform L | 9 | 9 | 9 | 9 |
| Fusiform R | 22 | 22 | 18 | 5 |
| Postcentral L | 53 | 40 | 40 | 18 |
| Postcentral R | 44 | 40 | 31 | 22 |
| Parietal Sup L | 31 | 22 | 22 | 14 |
| Parietal Sup R | 18 | 5 | 5 | 14 |
| Parietal Inf L | 9 | 14 | 18 | 9 |
| Parietal Inf R | 0 | 0 | 0 | 9 |
| SupraMarginal L | 27 | 22 | 22 | 18 |
| SupraMarginal R | 0 | 9 | 0 | 0 |
| Angular L | 5 | 5 | 5 | 9 |
| Angular R | 0 | 0 | 5 | 0 |
| **Precuneus L** | **57** | **27** | **44** | **18** |
| Precuneus R | 44 | 9 | 14 | 5 |
| Paracentral Lobule L | 44 | 70 | 40 | 22 |
| Paracentral Lobule R | 48 | 48 | 44 | 14 |
| Caudate L | 5 | 9 | 0 | 0 |
| Caudate R | 0 | 9 | 9 | 14 |
| Putamen L | 22 | 27 | 31 | 22 |
| Putamen R | 5 | 5 | 22 | 9 |
| Pallidum L | 5 | 5 | 18 | 9 |
| Pallidum R | 0 | 0 | 9 | 0 |
| Thalamus L | 9 | 9 | 18 | 9 |
| Thalamus R | 9 | 9 | 9 | 5 |
| Heschl L | 35 | 27 | 40 | 5 |
| **Heschl R** | **57** | **44** | **53** | **18** |
| **Temporal Sup L** | **61** | **66** | **53** | **22** |
| **Temporal Sup R** | **66** | **61** | **53** | **22** |
| **Temporal Pole Sup L** | **74** | **74** | **83** | **44** |
| **Temporal Pole Sup R** | **57** | **53** | **61** | **31** |
| **Temporal Mid L** | **70** | **74** | **92** | **57** |
| Temporal Mid R | 27 | 31 | 31 | 9 |
| Temporal Pole Mid L | 0 | 14 | 27 | 22 |
| Temporal Pole Mid R | 18 | 27 | 35 | 18 |
| Temporal Inf L | 9 | 0 | 14 | 0 |
| Temporal Inf R | 22 | 22 | 27 | 14 |
| Medial Prefront lower L | 44 | 48 | 35 | 35 |
| Medial Prefront lower R | 22 | 18 | 14 | 0 |
| **Medial Prefront upper L** | **74** | **70** | **70** | **74** |
| Medial Prefront upper R | 27 | 35 | 48 | 18 |
| Ant Insula L | 53 | 44 | 57 | 35 |
| Ant Insula R | 22 | 18 | 22 | 18 |
| Post Insula L | 27 | 14 | 22 | 0 |
| Post Insula R | 27 | 27 | 31 | 5 |
| Rostral ACC L | 0 | 0 | 0 | 0 |
| Rostral ACC R | 0 | 0 | 0 | 0 |
| Pregenual ACC L | 27 | 22 | 35 | 9 |
| Pregenual ACC R | 22 | 9 | 9 | 5 |
| **Dorsal ACC L** | **79** | **79** | **70** | **61** |
| **Dorsal ACC R** | **57** | **61** | **48** | **35** |
| **Posterior MCC L** | **83** | **87** | **70** | **70** |
| **Posterior MCC R** | **79** | **79** | **66** | **53** |
| 23d L | 35 | 9 | 27 | 9 |
| 23d R | 18 | 5 | 5 | 0 |
| dPCC L | 9 | 5 | 9 | 5 |
| dPCC R | 5 | 5 | 5 | 0 |
| vPCC L | 0 | 0 | 0 | 5 |
| vPCC R | 5 | 0 | 0 | 0 |
| TPJ L | 18 | 31 | 22 | 22 |
| TPJ R | 5 | 0 | 5 | 0 |
